# Division of labor in the nodule: Plant *GluTR*s fuel heme biosynthesis for symbiosis

**DOI:** 10.1093/plcell/koaf156

**Published:** 2025-06-12

**Authors:** Min-Yao Jhu, Raphael Ledermann

**Affiliations:** Assistant Features Editor, The Plant Cell, American Society of Plant Biologists; Crop Science Centre, Department of Plant Sciences, University of Cambridge, Cambridge CB3 0LE, UK; Department of Biology, University of Oxford, Oxford OX1 3RB, UK

Legume nodules are specialized, pink symbiotic organs where root cells and nitrogen-fixing rhizobia collaborate to support plant growth without synthetic fertilizers. The pink color is derived from the oxygen-carrying proteins leghemoglobins (Lbs) (Larrainzar et al. 2020). Nodules maintain an extremely low level of free oxygen, around 10,000 times lower than in ambient conditions (Batut and Boistard 1994), to protect the bacterial nitrogenase, the oxygen-sensitive enzyme responsible for nitrogen fixation. Even low oxygen levels irreversibly inactivate the iron-sulfur cluster cofactors within nitrogenase. Yet, nitrogen fixation is an energy-demanding process, requiring respiration to generate ATP and reducing equivalents. Lbs resolve this paradox by supplying just enough oxygen to the bacterial high-O_2_-affinity *cbb*_3_-type cytochrome *c* oxidase (Preisig et al. 1996) for respiration, without inactivating nitrogenase. The plant-derived Lbs require heme as a prosthetic group (Larrainzar et al. 2020) as do bacteroid proteins in the respiratory chain, but the primary source of nodule heme—whether from the plant or the rhizobia—has long been debated.

To investigate the source of nodule heme, Wang et al. (2025) conducted a genetic dissection and provided compelling evidence that *Lotus japonicus* heme biosynthesis is largely driven by plant glutamyl-tRNA reductases (GluTRs), particularly GluTR2 (Figure). GluTRs catalyze the rate-limiting step in the biosynthesis of 5-aminolevulinic acid (5-ALA), the universal precursor for tetrapyrroles, including heme (Kořený et al. 2022). To dissect the role of plant-derived 5-ALA, they generated *L. japonicus* CRISPR-Cas9 homozygous mutants for *GluTR2*, heterozygous knockouts of *GluTR1*, and transgenic plants overexpressing *FLU* (*Fluorescent in blue light*), a known negative regulator of GluTR activity (Meskauskiene et al. 2001). Because homozygous *glutr1* mutants were embryonic lethal, FLU overexpression (FLU-OE), driven by the *Lb2* promoter to target infected nodule cells (Wang et al. 2019), served as an effective strategy to suppress both GluTRs in infected cells. These genetic perturbations revealed that impairing host 5-ALA synthesis leads to a severe drop in nodule heme content and compromised symbiotic performance, underscoring the dominant role of host-derived GluTRs in coordinating heme production within nitrogen-fixing nodules.

**Figure.**
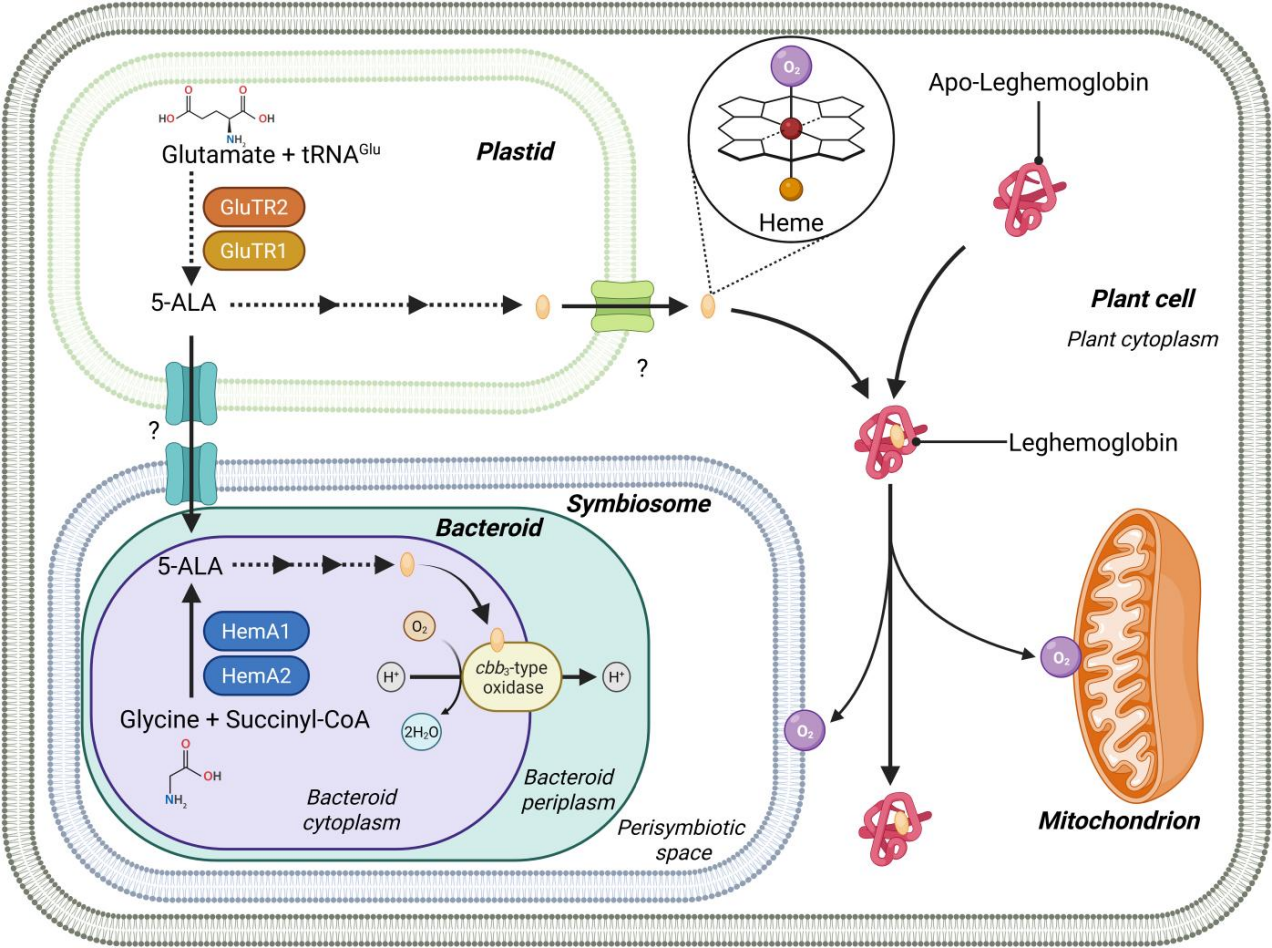
Coordinated heme biosynthesis in plant nodules. In *Lotus japonicus* nodules, both the plant and its rhizobial partner (*Mesorhizobium loti*) can make heme through a shared biosynthetic pathway that starts with the molecule 5-ALA. The plant produces 5-ALA from glutamate and tRNA^Glu^ in plastids using glutamyl-tRNA reductases (GluTR1 and GluTR2), while the rhizobia *M. loti* generate 5-ALA from glycine and succinyl-CoA using 5-aminolevulinate synthases (HemA1 and HemA2). From 5-ALA to heme, the pathway is nearly identical in both partners. Evidence suggests that 5-ALA may move from the plant to the rhizobia, and that heme could be transported to the cytoplasm, but how these molecules are moved between compartments is still unclear. Adapted Wang et al. (2025), Figure 6, using BioRender.com.

Interestingly, rhizobial *hemA1 hemA2* mutants, which cannot synthesize 5-ALA, had minimal impact on nodulation or nitrogen fixation. This is striking because 5-ALA is essential for heme biosynthesis, which suggests that the host supplies 5-ALA to its microbial partner. In contrast, rhizobial *hemN* mutants, defective in heme biosynthesis downstream of 5-ALA, were impaired in nodule colonization and nitrogen fixation. Together, this suggests that the plant not only produces its own heme but also supplies 5-ALA to its rhizobial partner. Transcriptomic analysis supported this view. Plant heme biosynthetic genes were strongly upregulated in nodules, while bacterial heme genes were broadly downregulated under symbiotic conditions. These findings reinforce the idea that bacteroids outsource biosynthetic labor, with the host acting as both a heme producer and supporting symbiotic metabolism via 5-ALA provision.

This outsourcing is not unique to 5-ALA. Rhizobia-legume symbioses are highly efficient because bacteroids, the symbiotic form of rhizobia within nodules, reside in a protected environment, which enables metabolic reprogramming for nitrogen fixation (Ledermann et al. 2021). Bacteroids depend on their hosts not only for nutrients but also for essential biosynthetic functions. Most rhizobia cannot fix nitrogen as free-living bacteria because they lack *nifV*, encoding the enzyme for biosynthesis of homocitrate, a nitrogenase cofactor, which is provided by the plant (Hakoyama et al. 2009). Bacteroids also downregulate branched-chain amino acid (valine, leucine, and isoleucine) biosynthesis, becoming entirely reliant on the host, a phenomenon termed symbiotic auxotrophy (Prell et al. 2009). The discovery that plants are the main source of 5-ALA in bacteroids aligns with this pattern of outsourcing biosynthesis to their hosts. 5-ALA is also the precursor of cobalamin (vitamin B_12_), a vital methyl donor. In *Sinorhizobium*, cobalamin auxotrophy disrupts symbiosis and nitrogen fixation by impairing methionine biosynthesis (Medina et al. 2009) and ribonucleotide reduction (Taga and Walker 2010), highlighting the importance of host-derived 5-ALA provision to bacteroids beyond heme biosynthesis.

Further insights into the regulation of Lbs came from FLU-OE plants inoculated with wild-type rhizobia, which exhibited ∼75% less nodule heme as well as ∼40% less Lb protein, which increasingly accumulated as apo-Lb, the inactive, heme-free form of leghemoglobin. This confirms that Lb biosynthesis is heme limited and dependent on plant GluTR activity. Intriguingly, despite reduced Lb protein levels, *Lb* mRNA increased by ∼47% in FLU-OE nodules, indicating post-transcriptional regulation. Although cysteine proteases are known to degrade Lbs during senescence (Yang et al. 2020), the expression of many cysteine protease genes was unchanged. However, several other proteases were upregulated and some protease inhibitors downregulated, suggesting a proteolytic shift favoring Lb degradation. These observations highlight the need for further investigation into the post-transcriptional and post-translational controls governing Lb stability. These findings reveal multiple layers of regulation in nodule heme biosynthesis—from transcriptional control to protein turnover—underscoring the complexity of host–microbe coordination.

This study reframes our understanding of symbiotic heme biosynthesis by placing plant GluTRs at the center of regulation. Yet several key questions remain: How is 5-ALA transported from plastids to bacteroids? What post-translational mechanisms fine-tune GluTR activity? How does the host manage heme levels to avoid cytotoxicity? Uncovering the transporters and regulatory circuits involved will not only deepen our knowledge of nodule biology but may also pave the way for engineering nitrogen-fixing capabilities in non-nodulating crops.

## Recent related articles in *The Plant Cell*

- Yu et al. (2024) showed that NODULE INCEPTION (NIN) directly activates *NAD1* through conserved cis-elements to regulate rhizobial accommodation in nodules of nitrogen-fixing clade plants.
- Drapek et al. (2024) demonstrated that gibberellin accumulates at nodule primordia and promotes nodule development in *Medicago truncatula*.
- Yu et al. (2023) identified *GmNAC039* and *GmNAC018* as key regulators of nodule senescence in soybean via activation of cysteine protease genes.

## Dive Curated Terms

The following phenotypic, genotypic, and functional terms are of significance to the work described in this paper:

FLU Gramene: AT3G14110
FLU Araport: AT3G14110

ATP CHEBI: CHEBI:15422
